# Supplementary figures and images for: Satellite Subgenomic Particles Are Key Regulators of Adeno-Associated Virus Life Cycle
Source: Viruses. 2021 Jun 21;13(6):1185. doi: 10.3390/v13061185 (PMC8235507; doi:10.3390/v13061185)

Supplementary Figure S1

A

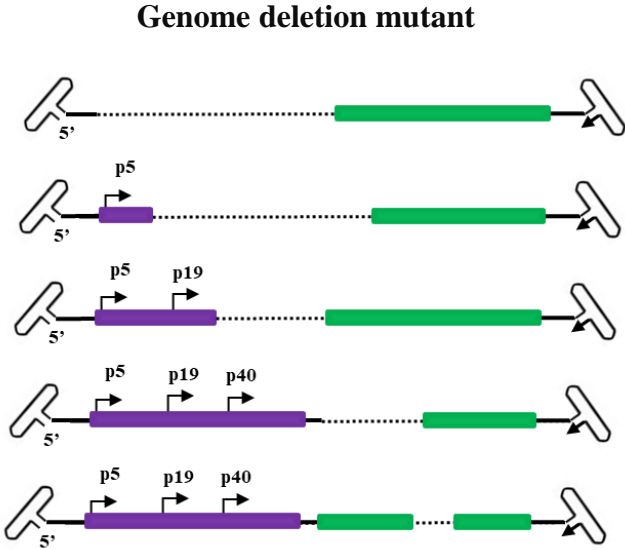

B

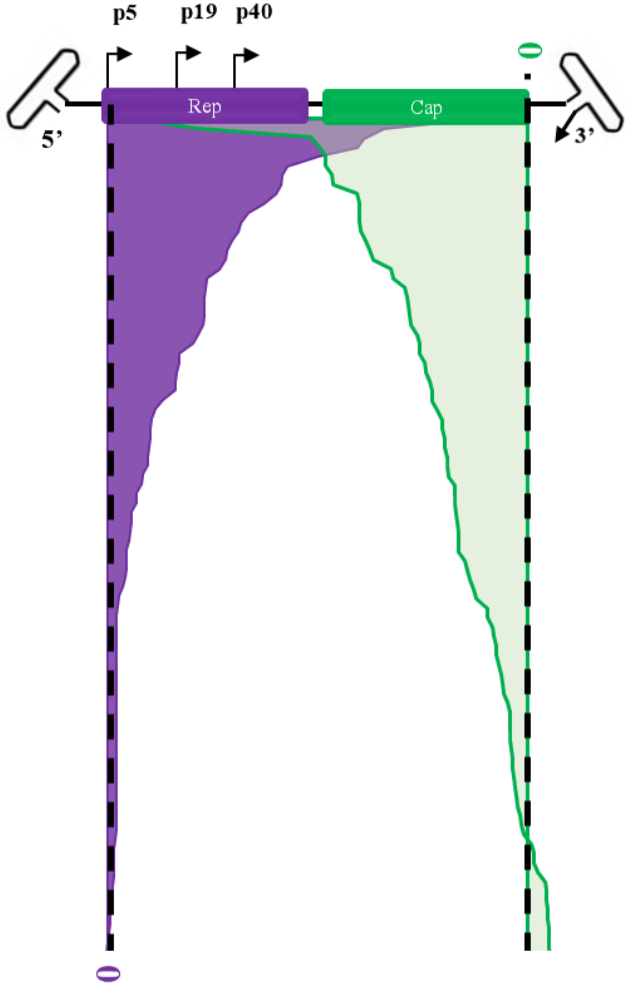

Supplementary Figure S2

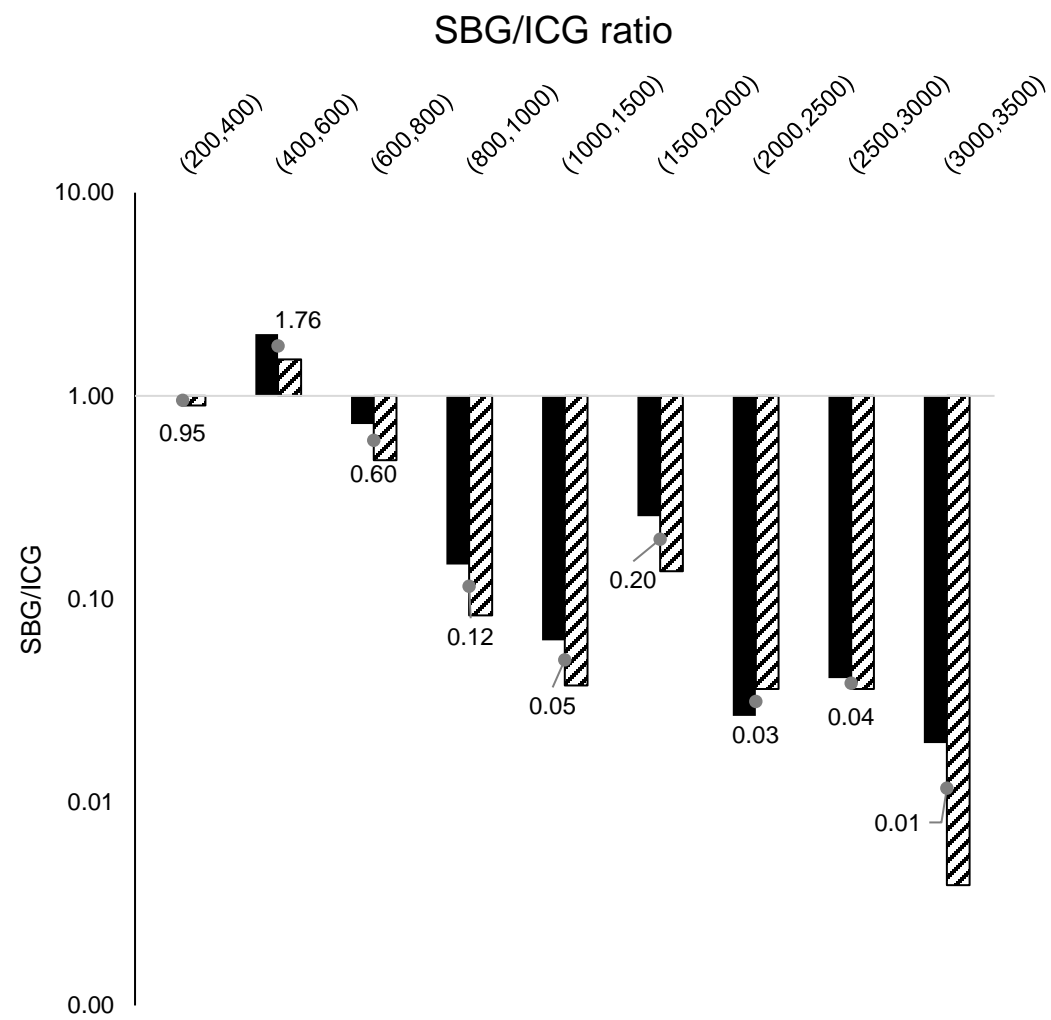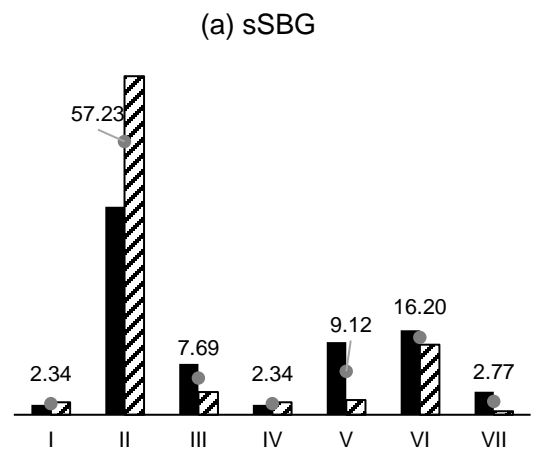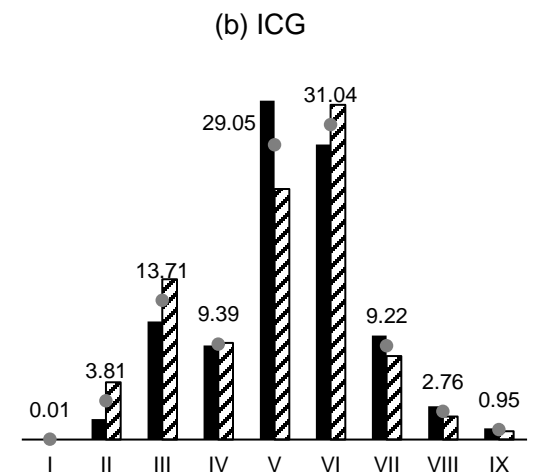

Supplementary Figure S3

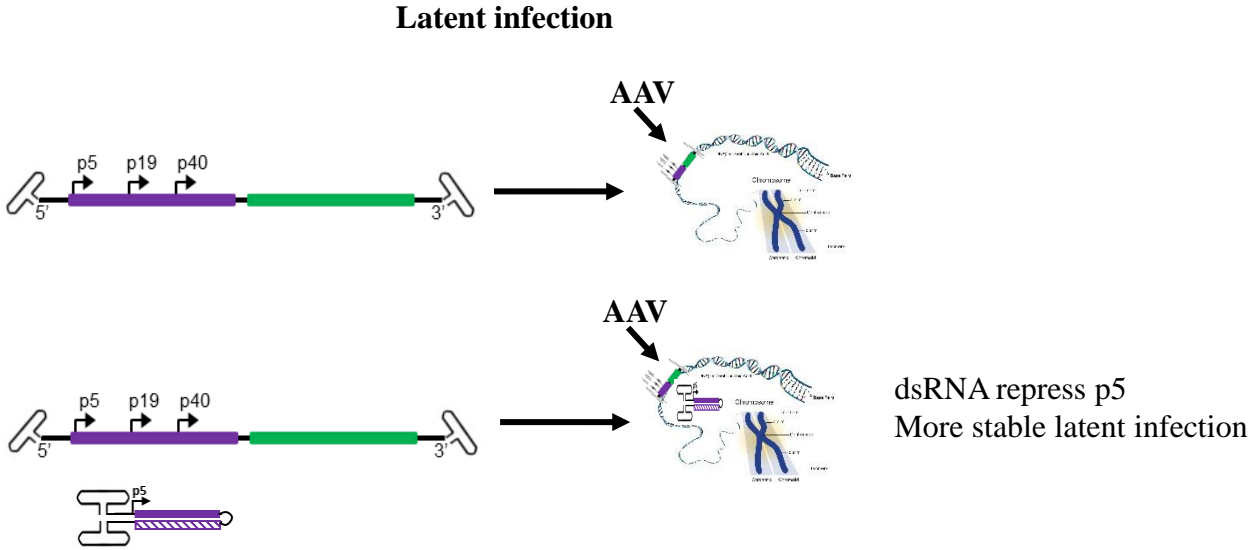

Supplement: Supplementary file 1 [file viruses-13-01185-s001.zip › viruses-1257755-supplementary.pdf]
